# Dyadic approach to post-stroke hospitalizations: role of caregiver and patient characteristics

**DOI:** 10.1186/s12883-019-1510-4

**Published:** 2019-11-04

**Authors:** Shilpa Tyagi, Gerald C. H. Koh, Nan Luo, Kelvin B. Tan, Helen Hoenig, David B. Matchar, Joanne Yoong, Angelique Chan, Kim En Lee, N. Venketasubramanian, Edward Menon, Kin Ming Chan, Deidre Anne De Silva, Philip Yap, Boon Yeow Tan, Effie Chew, Sherry H. Young, Yee Sien Ng, Tian Ming Tu, Yan Hoon Ang, Keng He Kong, Rajinder Singh, Reshma A. Merchant, Hui Meng Chang, Tseng Tsai Yeo, Chou Ning, Angela Cheong, Yu Li Ng, Chuen Seng Tan

**Affiliations:** 1 Saw Swee Hock School of Public Health, National University of Singapore, 12 Science Drive 2, #10-01, Singapore, 117549 Singapore; 2 Policy Research & Economics Office, Ministry of Health, Singapore, Singapore; 3 Physical Medicine and Rehabilitation Service, Durham VA Medical Centre, Durham, USA; 4 Program in Health Services and Systems Research, Duke-NUS Graduate Medical School, Singapore, Singapore; 5 Lee Kim En Neurology Pte Ltd, Singapore, Singapore; 6 Raffles Neuroscience Centre, Raffles Hospital, Singapore, Singapore; 7 St. Andrew’s Community Hospital, Singapore, Singapore; 8 Mount Alvernia Hospital, Singapore, Singapore; 9 National Neuroscience Institute, Singapore General Hospital campus, Singapore, Singapore; 10 Geriatric Medicine, Khoo Teck Puat Hospital, Singapore, Singapore; 11 St. Luke’s Hospital, Singapore, Singapore; 12 Department of Rehabilitation Medicine, National University Hospital, Singapore, Singapore; 13 Department of Rehabilitation Medicine, Changi General Hospital, Singapore, Singapore; 14 Department of Rehabilitation Medicine, Singapore General Hospital, Singapore, Singapore; 15 Department of Neurology, National Neuroscience Institute, Neurology, Tan Tock Seng Hospital, Singapore, Singapore; 16 Department of Rehabilitation Medicine, Tan Tock Seng Hospital, Singapore, Singapore; 17 Department of Medicine, Yong Loo Lin School of Medicine, National University of Singapore, Singapore, Singapore; 18 Department of Neurosurgery, National University Hospital, Singapore, Singapore

**Keywords:** Stroke, Healthcare utilization, Hospitalization, Caregiving, Family caregivers

## Abstract

**Aim:**

To study the association of caregiver factors and stroke patient factors with rehospitalizations over the first 3 months and subsequent 3–12 months post-stroke in Singapore.

**Methods:**

Patients with stroke and their caregivers were recruited in the Singapore Stroke Study, a prospective yearlong cohort. While caregiver and patient variables were taken from this study, hospitalization data were extracted from the national claims database. We used Poisson modelling to perform bivariate and multivariable analysis with counts of hospitalization as the outcome.

**Results:**

Two hundred and fifty-six patient with stroke and caregiver dyads (*N* = 512) were analysed, with patients having spouse (60%), child (29%), sibling (4%) and other (7%) as their caregivers. Among all participants, 89% of index strokes were ischemic, 57% were mild in severity and more than half (59%) of the patients had moderate or severe disability post-stroke as measured on the Modified Rankin Scale. Having social support in the form of a foreign domestic worker for general help of caregiver reduced the hospitalization rate over 3 months post-stroke by 66% (IRR: 0.342; 95% CI: 0.180, 0.651). Compared to having a spousal caregiver, those with a child caregiver had an almost three times greater rate of hospitalizations over 3–12 months post-stroke (IRR: 2.896; 95% CI: 1.399, 5.992). Higher reported caregiving burden at the 3-month point was associated with the higher subsequent rate of hospitalization.

**Conclusion:**

Recommendations include the adoption of a dyadic or holistic approach to post-stroke care provision by healthcare practitioners, giving due importance to both patients with stroke and their caregivers, integrating caregivers in the healthcare system to extend the care continuum to include informal care in the community and provision of timely support for caregivers.

## Introduction

Stroke is responsible for a significant global mortality burden with 16 million annual stroke cases resulting in 5.7 million deaths [1]. In addition to causing pain and suffering at an individual level, stroke also taxes the economies worldwide accounting for almost 4% of the direct healthcare costs in the developed nations [2]. Within Singapore, stroke along with other cerebrovascular diseases is one of the top ten causes of hospitalization [3], with average length of stay for acute care in a tertiary hospital for a stroke episode being about 7.7 days [4]. After stabilization in a tertiary hospital, patients with stroke may undergo intensive rehabilitation based on rehabilitation eligibility or be discharged to home or a step-down facility in the community. Rehospitalizations post-stroke are frequent, with the prevalence ranging from 18.8 to 49% [5–7]. Past efforts have identified stroke survivor’s age, functional status, stroke severity, comorbid status as some of the predictors of rehospitalizations [5, 7–9].

Stroke is a life changing event, not just for the patient but also for the family caregivers. With competing work, familial and personal obligations, an imbalance often occurs in the family caregiver’s capacity to care and the caregiving demands leading to caregiver role strain and the potential ripple effect on the outcomes of patients with stroke. Therefore, it is important to study post-stroke rehospitalization from a dyadic or holistic perspective, including both the caregiver and the patient factors. Among the studies adopting a dyadic approach, most of them involved heart failure patients [10, 11], dementia patients [12] or community dwelling elderly populations [13, 14]. Only few studies have adopted a dyadic perspective in studying rehospitalization post-stroke, with co-residing status [15], caregiver burden [16] and social support [17] as the main caregiver characteristics explored. Two of these studies were conducted in a Western setting and the findings may not be completely applicable to an Asian setting, with differences in the caregiving context [15, 17]. Moreover, both of these studies incorporated a single caregiver covariate in analysing the determinants of rehospitalization after stroke which may not be comprehensive enough to capture the role caregivers can play in such rehospitalizations. Chuang and colleagues [16] examined the association of caregiver determinants with rehospitalization over a short period of 1 month post-stroke in Taiwan. Their sample was limited to stroke survivors with functional impairments at discharge, which limited the generalizability of their findings to this sub-group of stroke survivors. To address these above mentioned gaps, we aimed to study the association of caregiver factors and patient factors with rehospitalizations over the first 3 months and subsequent 3–12 months post-stroke in Singapore.

## Methods

### Participants

Patients with stroke and their caregivers were recruited in the Singapore Stroke Study, a prospective cohort study with recruitment extending from December 2010 to September 2013 [18]. Recruitment was conducted in all five tertiary hospitals in Singapore during this period ensuring the representativeness of our sample. Eligible patients were Singaporeans or permanent residents 40 years and above, residing in Singapore for the next 1 year, with a recent stroke diagnosis (i.e. stroke symptoms occurring within 4 weeks prior to admission), stroke diagnosis made by a clinician and/or supported by brain imaging via CT or MRI and not globally aphasic. A caregiver could be an immediate or extended family member or friend, more than 21 years of age (the legal definition of adult in Singapore), providing care or assistance of any kind and taking responsibility for the patient (as recognized by the patient) and not fully paid for caregiving. The Singapore Stroke Study was approved by the National University of Singapore Institutional Review Board, SingHealth Centralized Institutional Review Board and the National Health Group Domain Specific Review Board. Written informed consent was obtained from both the patients and the caregivers.

### Data collection

Data were collected via face-to-face interviews with patients who had a stroke and their caregivers at baseline, 3-month and 12-month time points and via telephone interviews with caregivers at 6-month and 9-month time points. These interviews were conducted by a team of research assistants, who underwent a 3 days training to learn about the content and right method of administering the survey. The research assistants captured self-reported information from the participants under the broad clinical, social and financial domains. Efforts were taken to ensure good compliance, such as scheduling the interviews over the weekends or evenings of the weekdays to accommodate the participants’ busy schedule. We sent out reminders before each interview as well. Multiple attempts (upto 3) were made to reach out to participants before labelling them as uncontactable. We took steps to standardize the quality of data collection by training the research assistants in a consistent manner using video-recordings of initial rounds of training. The questionnaires were pilot tested on 40 participants from two sites and necessary changes were incorporated to finalize the data collection instruments.

For the current analysis, while dependent variables were extracted from the national claims database, independent variables were taken from the Singapore Stroke Study.

### Dependent variable (national claims database)

The dependent variable was counts of hospitalizations within the first 3 months following admission for stroke (excluding the index stroke related hospitalization) and the subsequent 3 to 12 months post-stroke. National claims database is a nation-wide database of healthcare utilization and associated expenditure maintained centrally by the Ministry of Health in Singapore. With the aid of a unique identification number allocated to all Singapore citizens and permanent residents, we linked our prospective cohort data with the healthcare data in the national claims database, achieving a match rate of more than 95%.

### Independent variables (caregiver factors)

Our primary independent variables were caregiver related, collected at the 3-month time point: socio-demographic variables (e.g. age, gender, ethnicity, marital status), caregiver relationship, comorbid conditions (self-reported by the caregivers), co-residing with the care recipient, caregiver-reported patient behavioural problems, caregiver burden (objective and subjective), family conflict (towards patient and caregiver), social support and caregiver-adopted care management/coping strategies (positive and negative). Caregiver relationship variable captured the relationship of the family caregiver to the patient with stroke and comprised of spousal, sibling, child and others as options. Others comprised of distant relatives and friends. Comorbid status variable was self-reported by caregivers as any health condition diagnosed since the last interview from a pre-designated list of 21 diseases (e.g., diabetes, hypertension and others). The total sum of reported health conditions was categorized as none, 1, 2 or 3 and more reported comorbid conditions. Since our intention was to study the longitudinal effects of caregiving over the observation period, we captured the self-reported chronic ailments that the caregivers developed during this follow-up period, instead of capturing the pre-existing chronic conditions that the caregiver may have had. We used the Revised Memory and Behavioural Problem Checklist to capture the variable of caregiver reported problematic behaviour of patients with stroke [19]. Responses were recorded on a 5-point Likert scale: 0 = never, 1 = not in the past week, 2 = 1 to 2 times per week, 3 = 3 to 6 times per week and 4 = daily or more often. We summated the total score across three broad domains of memory-related, disruptive and depressive behavioural problems with the score range being 0–28 across each domain. Caregiver burden variable was captured using two instruments: Oberst Caregiving Burden Scale (for objective burden estimate) and Zarit’s Burden Interview (for subjective burden estimate). Oberst caregiving burden scale, reported to have good psychometric properties in stroke caregivers, was used to capture the amount of time spend on different caregiving activities by the caregiver. Total of 15 items were scored on a Likert scale from 1 = none to 5 = a great amount and the total score ranged from 15 to 75 [20]. Caregiver’s appraisal of caregiving impact was captured by the Zarit’s Burden Interview, which involved asking caregivers to rate how often they felt several negatively phrased questions related to their caregiving role [21]. Validated previously in Singapore [22], we used the abbreviated 12-item version for the current study with a total score ranging from 0 to 48. Social support variable was captured at two levels in accordance with the guidance given by Pearlin and colleagues [23]. Firstly, social support comprised of the physical network of caregiver, and for the current study, we used two categorical variables documenting the presence of foreign domestic worker (FDW) or paid helper for general household chores and specifically for stroke patient care respectively. A FDW is “*a stay-in migrant female waged domestic worker attached to one employer and works for only a single household, under Singapore’s strict legal permit system*.” [24] Ministry of Manpower in Singapore mandates that they stay and provide services within the homes of their employers [25]. They hold an important position in the context of increasingly aging Singapore population. They lend support to family members across a range of activities including household chores to providing care to their loved ones. FDWs are paid a fixed monthly salary along with provision of a room in the household they work [26]. Second type of social support measured was the perceived social support, using Pearlin’s 8-item perceived social support instrument (Cronbach’s alpha of 0.87) with responses captured on a 4-point Likert scale and a total score ranging from 4 to 32. We used the revised dementia management strategies instrument to capture care management or coping strategies by caregivers of patients with stroke. The 20-item instrument version has been validated in Singapore [27] and records responses to the frequency of adopted strategy on 5-point Likert scale: 1 = never, 2 = seldom, 3 = sometimes, 4 = often and 5 = most of the time. The instrument comprises of two subscales of positive and negative dimensions, with good reported internal consistency in Singapore population (Cronbach’s alpha 0.89 and 0.87 respectively) [27]. We summated the total score across these two dimensions of positive (Range: 12 to 60) and negative (Range: 8 to 40) care management strategies. We measured family caregiving conflict adopting 8 items used by Pearlin and colleagues [23], under two broad areas of attitudes and actions involving the patient and the caregiver. Higher scores denoted more family caregiving conflicts.

### Independent variables (patient factors)

We considered the following patient variables collected at baseline: socio-demographic variables comprising of age, gender, ethnicity, marital status; ward class; comorbid condition (captured using Charlson Comorbidity Index) [28]; stroke type (ischemic or non-ischemic); recurrent stroke, stroke severity (measured using National Institute of Health Scale, NIHSS) [29], level of disability (measured using Modified Rankin Scale, mRS) [30], cognitive impairment (using the Mini-Mental State Examination, MMSE) [31] and discharge destination. Ward class denoted the ward category where the patient with stroke stayed during the index hospitalization. In Singapore, government provides subsidies for inpatient stay in the tertiary care setting based on a tiered system, to make healthcare affordable for all. Based on a means tested eligibility criteria, patients can be admitted to A, B1, B2 or C ward category being entitled to increasing level of subsidies. While the quality of care across these wards is similar, for example, team-based care led by a specialist, they differ in the level of amenities provided to the patient. For instance, ward class C may include 8 to 10 beds with natural ventilation and can be upto 80% subsidized. While ward class A may include a single-bed, be air-conditioned with 0% subsidy. This categorization of ward class is implemented across all clinical specialties or departments within the tertiary care setting. For current analysis, we binarized this ward class variable into subsidized and non-subsidized ward class [32]. For scales with significant (> 10) number of missing cases (NIHSS, MMSE, Revised memory and behaviour checklist), we used the person mean substitution approach to impute for missing values for cases with less than half the constituent items missing [33].

### Data analysis

Univariate analysis was performed to describe our sample of stroke patient-caregiver dyads. Bivariate analysis was performed to examine the associations between caregiver and patient factors and risk of hospitalization post-stroke. Independent variables (caregiver and patient factors) having *p*-value < 0.1 in the bivariate analyses were chosen as potential predictors for the multivariable regression model. Using these potential predictors, a backward variable selection approach was conducted to identify the most parsimonious model by removing the most insignificant variable at each model building iteration step until all the variables that remained in the model had a p-value< 0.05 (except for age, gender, ethnicity and ward class of the patient with stroke which we opted to retain in the model apriori to standardize for socio-demographic variables). We used Poisson modelling to perform the bivariate analyses and the backward variable selection approach to regression. With the most parsimonious adjusted model, we assessed for over-dispersion and excessive zeroes using alpha statistic and Voung test respectively [34, 35] and applied the appropriate regression model accordingly. We reported the unadjusted and adjusted incidence rate ratio (IRR) estimates with 95% confidence intervals (CI). Separate models were run for the following two time periods: first 3 months post-stroke and subsequent 3–12 months post-stroke. Significance level was set at 5%. We conducted a sensitivity analysis to examine the effect of the length of stay during the index stroke episode on our findings for the early post-stroke period.

## Results

**Fig. 1:**
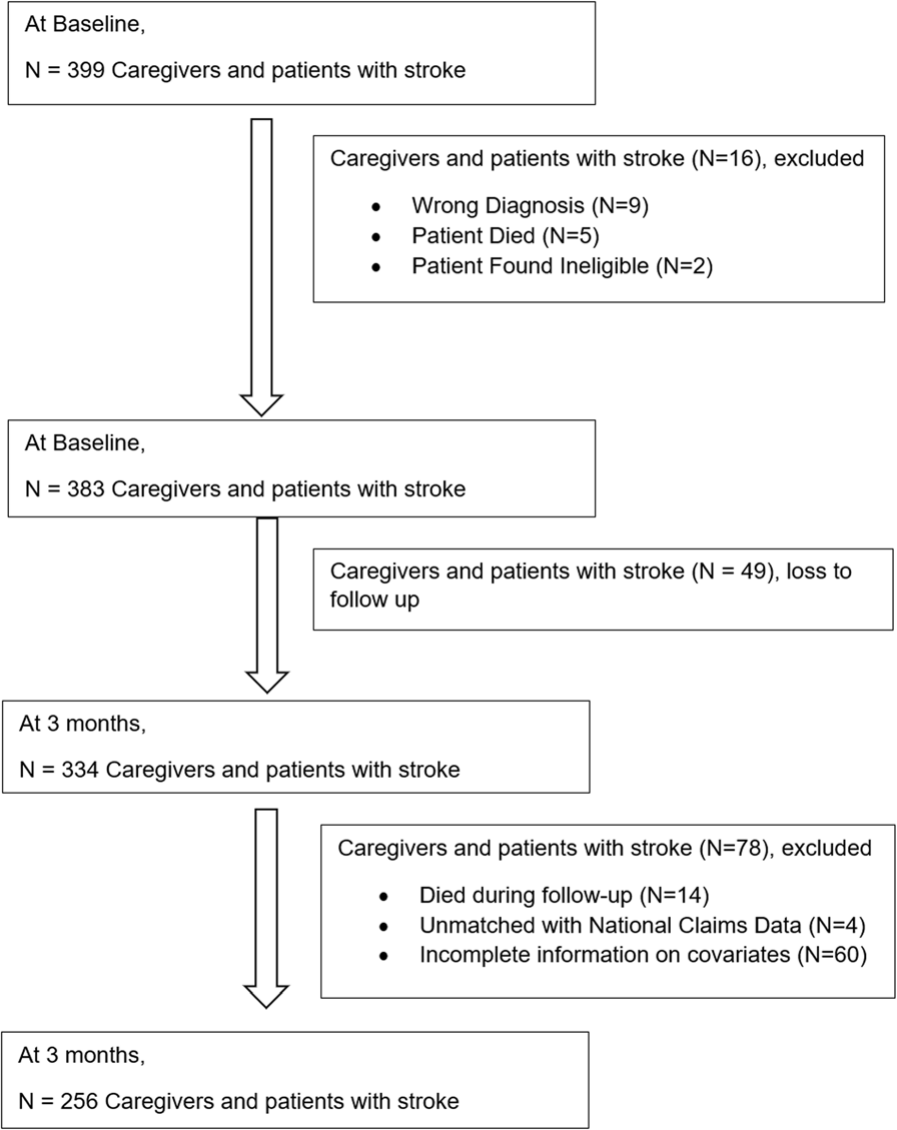
Study Flowchart

Two thousand nine hundred thirty one patients with stroke were identified and assessed for eligibility, out of which 661 patients with stroke were recruited at baseline. We assessed the caregivers of recruited 661 patients for eligibility to participate and recruited 399 caregivers at baseline. Two hundred and fifty-six stroke patient-caregiver dyads were available for the current analysis, after merging across both databases and exclusion of patients with deaths within the follow up period. Please refer to Fig. 1 for the study flowchart. While 64% of the patients were male, the majority (76%) of the caregivers were females. The average age of the patients was 62 years, while that of the caregivers was 50 years. The majority of the patients with stroke and their caregivers were married. Among all the participants, 89% of the index strokes were ischemic, and the index stroke was a recurrent event for 16% of the participants. One-fourth of the patients with stroke had MMSE score ranging between 18 and 23 at the baseline, and more than half (59%) had moderate or severe disability post-stroke as measured on the mRS. Patients could have a spouse (60%), a child (29%), a sibling (4%) or others (7%) as their caregivers with the majority of the caregivers (89%) residing with the patient. Please refer to Tables 1 for more detail.

**Table 1:** Descriptive characteristics of caregivers and patients

	No. (%) unless otherwise stated	
CAREGIVER^*^ FACTORS		
Age (in years)	Mean (SD)	50.0 (13.2)
Gender	Male	61 (24)
	Female	195 (76)
Ethnicity	Chinese	147 (57)
	Non-Chinese	109 (43)
Marital Status	Married	201 (79)
	Single	55 (21)
Caregiver relationship	Spouse	155 (60)
	Child	74 (29)
	Sibling	10 (4)
	Others	17 (7)
Comorbid conditions	None	153 (60)
	1	57 (22)
	2	24 (9)
	≥3	22 (9)
Co-residing with patient	Yes	227 (89)
	No	29 (11)
Caring for multiple care recipients	Yes	106 (41)
	No	150 (59)
Revised memory and behavior checklist		
Memory problems	Mean (SD)	5.1 (6.0)
Depressive behavior problems	Mean (SD)	3.2 (4.9)
Disruptive behavior problems	Mean (SD)	2.7 (3.6)
Caregiver burden		
Oberst Caregiving Burden Scale	Mean (SD)	31.8 (12.7)
Zarit Burden Interview	Mean (SD)	8.8 (7.9)
Family conflict		
Attitude towards patient	Mean (SD)	11.4 (4.5)
Attitude towards caregiver	Mean (SD)	11.6 (4.4)
Social support (instrumental)		
FDW for general help	Yes	208 (81)
	No	48 (19)
FDW for stroke patient	Yes	33 (13)
	No	223 (87)
Social Support (perceived)	Mean (SD)	26.4 (4.9)
Care management strategies		
Positive strategies	Mean (SD)	34.5 (10.8)
Negative strategies	Mean (SD)	11.1 (4.6)
PATIENT^#^ FACTORS		
Age (in years)	Mean (SD)	61.8 (10.5)
Gender	Male	165 (64)
	Female	91 (36)
Ethnicity	Chinese	149 (58)
	Non-Chinese	107 (42)
Marital Status	Married	206 (80)
	Single	50 (20)
Ward Class	Unsubsidized	21 (8)
	Subsidized	235 (92)
Charlson Comorbidity Index (during stroke onset)	1–3	52 (20)
	4–6	162 (64)
	≥ 7	42 (16)
Stroke type	Ischemic	227 (89)
	Non-ischemic	29 (11)
Recurrent stroke	Yes	42 (16)
	No	214 (84)
National Institute of Health Scale	Mild (0–4)	145 (57)
	Moderately severe (5–14)	97 (38)
	Severe (15–24)	14 (5)
Modified Rankin Scale	No or slight disability (0–2)	105 (41)
	Moderate or severe disability (3–5)	151 (59)
Mini-Mental State Examination	No (24–30)	147 (57)
	Mild (18–23)	64 (25)
	Severe (1–17)	45 (18)
Discharge to Community Hospital or step-down facility	Yes	65 (25)
	No	191 (75)
Centre for Epidemiological Studies Depression Scale	Mean (SD)	6.3 (5.6)

^*^Variables collected at 3-month time point; ^#^Variables collected at baseline

Abbreviations: *No.* Number, *SD* Standard deviation, *FDW* Foreign domestic worker

Table 2 details the results of the association of the caregiver and the patient characteristics with the rate of hospitalization across the early (0–3 months) post-stroke period. The variables retained in the final adjusted model of hospitalization in the first 3 months post-stroke were the presence of a FDW for general help and recurrent stroke. Having a FDW for general help of the caregiver reduced the expected rate of hospitalization over 3 months post-stroke by 66% as compared to those having no FDW (IRR: 0.342; 95% CI: 0.180, 0.651). For the stroke survivors with index stroke being a recurrent one, the expected rate of hospitalization was twice that of the stroke survivors with index stroke being the first one (IRR: 2.099; 95% CI: 1.136, 3.877). The interpretation of our findings remained unchanged after the addition of length of stay for the index stroke episode in the final adjusted model for the early post-stroke period. (Please refer Additional file 1).

**Table 2:** Association of caregiver and patient characteristics with rehospitalization 0–3 months post-stroke

	Reference category (if applicable)	IRR (95% CI)	*P*-value	aIRR^a^ (95% CI)	*P*-value
CAREGIVER FACTORS					
Age (in years)		0.996 (0.975, 1.017)	0.708		
Gender	Male	0.665 (0.367, 1.204)	0.178		
Ethnicity	Non-Chinese	0.944 (0.540, 1.650)	0.839		
Marital Status	Single	1.247 (0.606, 2.565)	0.549		
Caregiver relationship	Spouse		0.510		
Child		1.300 (0.722, 2.341)			
Sibling		1.069 (0.255, 4.480)			
Others		0.314 (0.043, 2.308)			
Comorbid Conditions	None		0.103		
1		0.395 (0.154, 1.009)			
2		1.500 (0.694, 3.240)			
≥ 3		0.614 (0.188, 1.998)			
Co-residing with patient	No	0.671 (0.315, 1.429)	0.300		
Caring for multiple care recipients	No	1.533 (0.880, 2.670)	0.131		
Memory problems		1.020 (0.977, 1.064)	0.367		
Depressive behavior problems		1.043 (0.995, 1.092)	0.077		
Disruptive behavior problems		1.047 (0.983, 1.114)	0.152		
Oberst Caregiving Burden Scale		1.011 (0.991, 1.032)	0.289		
Zarit Burden Interview		1.005 (0.971, 1.040)	0.778		
Family conflict - Attitude towards patient		0.958 (0.902, 1.017)	0.157		
Family conflict - Attitude towards caregiver		0.956 (0.899, 1.016)	0.146		
Social support - FDW for general help	No	0.410 (0.230, 0.731)	0.002	0.342 (0.180, 0.651)	0.001
Social support - FDW for stroke patient	No	2.374 (1.262, 4.467)	0.007		
Social Support (perceived)		1.020 (0.962¸ 1.081)	0.508		
Care management strategies - Positive		1.011 (0.985, 1.037)	0.429		
Care management strategies - Negative		1.041 (0.988, 1.096)	0.134		
PATIENT FACTORS					
Age (in years)		0.982 (0.955, 1.009)	0.180		
Gender	Male	1.425 (0.815, 2.490)	0.214		
Ethnicity	Non-Chinese	1.277 (0.717, 2.274)	0.407		
Marital Status	Single	0.769 (0.402, 1.471)	0.427		
Ward Class	Unsubsidized	0.804 (0.319, 2.026)	0.644		
CCI (during stroke onset)	1–3		0.495		
4–6		1.244 (0.572, 2.706)			
≥ 7		1.702 (0.685, 4.232)			
Stroke type	Non-ischemic	0.405 (0.211, 0.774)	0.006		
Recurrent stroke	No	2.184 (1.193, 3.998)	0.011	2.099 (1.136, 3.877)	0.018
National Institute of Health Scale	Mild (0–4)		0.022		
Moderately severe (5–14)		1.427 (0.785, 2.595)			
Severe (15–24)		3.295 (1.408, 7.714)			
Modified Rankin Scale	No or slight disability (0–2)				
Moderate or severe disability (3–5)		1.979 (1.052, 3.723)	0.034		
Mini-Mental State Examination	No cognitive impairment (24–30)		0.197		
Mild cognitive impairment (18–23)		1.398 (0.719, 2.717)			
Severe cognitive impairment (1–17)		1.846 (0.935, 3.645)			
Discharge to Community Hospital (step-down facility)	No	1.514 (0.843, 2.718)	0.165		
Centre for Epidemiological Studies Depression Scale		1.020 (0.971, 1.072)	0.427		

Abbreviations: *IRR* Incidence rate ratio, *aIRR* Adjusted incidence rate ratio, *CI* Confidence interval, *CCI* Charlson Comorbidity Index, *FDW* Foreign domestic worker

^a^Model adjusted for age, gender, ethnicity and ward class of the patient

Table 3 details the results of the association of the caregiver and the patient characteristics with the rate of hospitalization across the late (3–12 months) post-stroke period. The bivariate association of the comorbid status of stroke survivors, measured on Charlson Comorbidity Index, with hospitalization in the late post-stroke period with statistically significant, but this variable was not retained in the final adjusted model. Similarly, the bivariate association of self-reported comorbid conditions of caregivers with hospitalization in the late post-stroke period was statistically significant, but this variable was not retained in the final adjusted model. The variables retained in the final adjusted model of hospitalization 3–12 months post-stroke were caregiver relationship and the subjective caregiving burden, measured using Zarit’s Burden Interview. Compared to having a spousal caregiver, those having a child caregiver had almost three times greater rate of hospitalizations over 3–12 months post-stroke (IRR: 2.896; 95% CI: 1.399, 5.992). Higher reported caregiving burden at the 3-month time point was associated with the higher subsequent rate of hospitalization. With every 1 unit increase in the Zarit Burden Interview score, the rate of hospitalization increased by a factor of 1.038 (95% CI: 1.002, 1.076).

**Table 3:** Association of caregiver and patient characteristics with rehospitalization 3–12 months post-stroke

	Reference category (if applicable)	IRR (95% CI)	*P*-value	aIRR^a^ (95% CI)	*P*-value
CAREGIVER FACTORS					
Age (in years)		0.983 (0.968, 0.998)	0.028		
Gender	Male	1.043 (0.640¸ 1.698)	0.866		
Ethnicity	Non-Chinese	0.792 (0.525, 1.195)	0.266		
Marital Status	Single	0.649 (0.414, 1.017)	0.059		
Caregiver relationship	Spouse		<0.001		0.032
Child		2.957 (1.906, 4.588)		2.896 (1.399, 5.992)	
Sibling		1.368 (0.420, 4.453)		0.952 (0.193, 4.687)	
Others		1.609 (0.676, 3.832)		1.435 (0.416, 4.953)	
Comorbid Conditions	None		<0.001		
1		0.842 (0.480, 1.477)			
2		2.500 (1.491, 4.193)			
≥ 3		0.545 (0.197, 1.509)			
Co-residing with patient	No	0.841 (0.458, 1.544)	0.576		
Caring for multiple care recipients	No	1.726 (1.142, 2.608)	0.010		
Memory problems		1.026 (0.995, 1.058)	0.106		
Depressive behavior problems		1.026 (0.989, 1.065)	0.173		
Disruptive behavior problems		0.999 (0.944, 1.058)	0.986		
Oberst Caregiving Burden Scale		1.021 (1.006, 1.036)	0.006		
Zarit Burden Interview		1.024 (1.001, 1.048)	0.039	1.038 (1.002, 1.076)	0.040
Family conflict - Attitude towards patient		0.966 (0.923, 1.010)	0.126		
Family conflict - Attitude towards caregiver		0.975 (0.931, 1.020)	0.271		
Social support - FDW for general help	No	0.469 (0.303, 0.726)	0.001		
Social support - FDW for stroke patient	No	2.703 (1.715, 4.260)	<0.001		
Social Support (perceived)		1.016 (0.973, 1.060)	0.478		
Care management strategies - Positive		1.017 (0.998, 1.037)	0.085		
Care management strategies - Negative		0.980 (0.934, 1.028)	0.408		
PATIENT FACTORS					
Age (in years)		1.026 (1.006, 1.046)	0.010		
Gender	Male	1.624 (1.076, 2.451)	0.021		
Ethnicity	Non-Chinese	0.703 (0.466, 1.060)	0.092		
Marital Status	Single	0.984 (0.588, 1.649)	0.952		
Ward Class	Unsubsidized	0.491 (0.278, 0.869)	0.014		
CCI (during stroke onset)	1–3		0.004		
4–6		1.049 (0.588, 1.870)			
≥ 7		2.229 (1.186, 4.189)			
Stroke type	Non-ischemic	1.035 (0.536, 1.996)	0.919		
Recurrent stroke	No	1.625 (1.005, 2.625)	0.047		
National Institute of Health Scale	Mild (0–4)		0.177		
Moderately severe (5–14)		1.325 (0.861, 2.039)			
Severe (15–24)		1.883 (0.887, 4.000)			
Modified Rankin Scale	No or slight disability (0–2)				
Moderate or severe disability (3–5)		2.318 (1.423, 3.775)	0.001		
Mini-Mental State Examination	No cognitive impairment (24–30)				
Mild cognitive impairment (18–23)		2.728 (1.704, 4.365)			
Severe cognitive impairment (1–17)		2.144 (1.236, 3.717)			
Discharge to Community Hospital (step-down facility)	No	1.374 (0.884, 2.136)	0.157		
Centre for Epidemiological Studies Depression Scale		1.040 (1.007, 1.074)	0.017		

Abbreviations: *IRR* Incidence rate ratio, *aIRR* Adjusted incidence rate ratio, *CI* Confidence interval, *CCI* Charlson Comorbidity Index, *FDW* Foreign domestic worker

^a^Model adjusted for age, gender, ethnicity and ward class of the patient

## Discussion

We are among the first to report that caregiver factors, such as caregiver burden, social support and caregiver relationship with the stroke survivor are significantly associated with rehospitalization after stroke, thus highlighting the importance of considering the stroke patient-caregiver dyad when providing post-stroke care. Adopting an approach to support the stroke patient-caregiver dyad can involve policy makers, health and social care providers and community partners holistically planning, implementing and evaluating interventions/programs by involving both the patients with stroke and their caregivers. The terminology and approach of considering the care recipient-caregiver dyads across various outcomes has been reported previously in both stroke [36–38] and non-stroke populations [39–41]. There is evidence that better outcomes are associated with active involvement of stroke survivors and their caregivers in interventions targeting stroke survivor outcomes. The authors broadly categorized the interventions into skill-building (e.g., problem solving, stress management), psycho-educational (e.g., information provision on warning signs of stroke) and support interventions, with interventions including skill-building and psycho-educational elements showing more promise than psycho-educational interventions alone [36]. We also demonstrated that different caregiver factors were significantly associated with rehospitalization across the early (0–3 months) and the late post-stroke period (3–12 months), highlighting the need for a tailored approach based on both caregiver factors and time considerations.

We reported that the presence of social support, in the form of a FDW for general help, was associated with a decreased rate of rehospitalization in the early post-stroke period. With transition from hospital to home being stressful for the patients and their caregivers, lack of social support may make it difficult for the caregiver to attend to the needs of patient with stroke in a timely manner due to competing commitments. This could possibly explain the delayed presentation in an acute care setting instead of earlier presentation in an appropriate setting. In concordance with our findings, a US based study on post-stroke rehospitalizations within 3 months of discharge reported that the patients having lower social support were 2.28 times more likely to be hospitalized as compared to those with high social support [17]. The association between social support and hospitalization is observed among other populations as well [11, 42, 43]. Though the manner in which social support was conceptualized differed across the different stroke and non-stroke studies, there was an overarching association of social support with reduced use of acute healthcare services. Our finding highlights the importance of supporting the caregivers in the community during the early post-stroke period to navigate the new caregiving role along with balancing other competing commitments. This social support from paid helpers or other family members may provide them the needed buffer to attain caregiving competency and adjust with changes related to this new role.

Caregiver burden is conceptually complex resulting from multiple patient and caregiver factors. In our study, subjective caregiver burden reported over the early post-stroke period was associated with an increased rate of rehospitalization in the late post-stroke period. A possible explanation could be that burdened caregivers may not be able to cope and care adequately for the patient with stroke, and the quality of care may be compromised. Subsequently, the conditions that could have been mitigated at earlier stages end up in the more expensive inpatient setting. Another possible explanation could be patients who are more likely to be readmitted are harder to care for or have more complications post-stroke, which may contribute towards the caregivers feeling more burdened. In concordance with our results, another study involving community dwelling older persons reported a positive association between the subjective caregiver burden at baseline and hospitalization over 3 years. Interestingly, this association between caregiver burden and hospitalization observed in the older persons did not hold in the subgroup using the respite services [13]. In other words, utilization of respite services modified the association between caregiver burden and hospitalization, supporting the possibility that such services may be useful to the caregivers in coping and adapting. Strengthening of such respite services in our context may be an avenue worth exploring to support the caregivers and alleviate the caregiver burden. Respite services in Singapore are mainly offered as inpatient or centre-based services for longer and shorter time periods, respectively [44]. With limited information available on the respite services utilization by the caregivers of patients who had a stroke, future research efforts should explore such utilization patterns and recommend ways to increase uptake of such services.

We found the caregiver relationship with stroke survivors to be associated with rehospitalization in the late post-stroke period. Wolff and colleagues reported a significant association between caregiver relationship with their care recipients and ever being hospitalized, with a lower percentage of spousal caregivers (as compared to child and others) in ever hospitalized group (*p* = 0.0325), but they did not distinguish whether those hospitalizations occurred in the early or the late period [14]. With limited prior research exploring the role of caregiver relationship in hospitalization post-stroke, our study fills an important gap. However, we acknowledge the importance of teasing out specific characteristics and the circumstances that vary across caregivers related in different capacities with the stroke survivor to further explain this observed association.

Our findings suggest that caregivers are an integral part of the stroke survivors’ post-acute care journey in the community. Efforts directed at stabilizing the caregivers in their caregiving role in the community may influence, and to some extent, may reduce the stroke survivors’ acute healthcare utilization post-stroke. By stabilization, we imply the provision of specific support to the stroke patient-caregiver dyad at appropriate times. Revisiting the scope of the caregiver’s role is a pre-requisite to determining their needs and providing adequate support. A recent review suggested viewing caregivers in multiple roles, as stakeholders in the recovery process, legitimate clients with their own needs and as advocates for their care recipients [45]. On the healthcare front, physicians can adopt a dyadic approach to risk assessment and stratification of patient with stroke and their caregivers, and calibrate the intensity of support and face-time with the health professionals based on the stratum of need, ensuring dyads can thrive in the community and avoid hospital visits [46]. Since we found support from FDW with general household tasks in the early post-stroke period was associated with reduced hospitalizations, the caregivers might benefit from extended support during this period. Further, giving more visibility to the stroke support organizations in the community and sharing caregiving experiences may help new caregivers adapt better. Policy level changes should focus on empowering the caregivers to fulfil their role successfully. Respite care services should be well-developed, culturally appropriate, and easily accessible to ensure the caregiver’s mental and physical well-being.

Our study has some limitations. Since our primary focus was on the caregiver determinants of rehospitalizations, our study does not provide reasons for such rehospitalizations. Future research efforts can qualitatively explore the reasons and circumstances of acute healthcare consumption post-stroke. Secondly, while we can comment about the temporality across caregiver factors and rehospitalizations over the late post-stroke period, we are limited to make such inferences over the early post-stroke period. We excluded those patients with stroke who died (*N* = 5) during the follow-up period of 1 year, as we wanted to examine the association of caregiver factors with the hospitalizations over this follow-up period. Therefore, our results will be generalizable to patients with stroke who survive the first year post-stroke.

Our study also has several strengths. It is among the first to illustrate how caregiver factors are associated with rehospitalizations after a stroke. Further, we demonstrated that caregiver determinants of rehospitalization differ across the early and late post-stroke period. In many healthcare utilization studies, caregiver factors are missing since such factors are not fully captured in the administrative records generally used for such studies. We had the unique opportunity to comprehensively explore caregiver and stroke patient characteristics, thus enabling us to holistically understand the clinical, socio-demographic, and caregiver determinants of hospitalization post-stroke. Using two data sources, one of which was the national claims database, enabled us to capture the rehospitalizations objectively without being affected by any loss to follow up beyond first 3 months.

## Conclusion

Our study demonstrated that caregiver burden, caregiver relationship with the stroke survivor and social support are significantly associated with rehospitalization post-stroke. Recommendations include adoption of a dyadic or holistic approach to studying hospitalizations after stroke including both the stroke survivor and the caregiver factors. On a practice front, focusing on integrating caregivers in the healthcare system to extend the continuum of care from formal to informal care in the community and mobilizing resources towards provision of timely support for the caregivers.

## Supplementary information

**Additional file 1.** Sensitivity analysis.

## Data Availability

The datasets used and analysed during the current study are available from the corresponding author on reasonable request.

## Abbreviations

FDW: Foreign domestic worker
IRR: Incidence rate ratio
MMSE: Mini-Mental State Examination
mRS: Modified Rankin Scale
NIHSS: National Institute of Health Scale
