# Supplementary material for: Dyadic approach to post-stroke hospitalizations: role of caregiver and patient characteristics
Source: BMC Neurol. 2019 Nov 4;19:267. doi: 10.1186/s12883-019-1510-4 (PMC6829975; doi:10.1186/s12883-019-1510-4)
Supplement: Supplementary file 1 — Additional file 1. Sensitivity analysis. [file 12883_2019_1510_MOESM1_ESM.pdf]

Sensitivity Analysis

Comparison of effect estimates of association of caregiver and patient characteristics with rehospitalization 0-3 months post-stroke across the final adjusted model without and with addition of length of stay for index stroke episode.

	**Reference category (if applicable)**	**aIRR* (95% CI)**	***P*-value**	**aIRR# (95% CI)**	***P*-value**
**CAREGIVER FACTORS**					
Age (in years)					
Gender	Male				
Ethnicity	Non-Chinese				
Marital Status	Single				
Caregiver identity	Spouse				
Adult-child					
Sibling					
Others					
Comorbid Conditions	None				
1					
2					
≥3					
Co-residing with patient	No				
Caring for multiple care recipients	No				
Memory problems					
Depressive behavior problems					

Disruptive behavior problems					
Oberst Caregiving Burden Scale					
Zarit Burden Interview					
Family conflict - Attitude towards patient					
Family conflict - Attitude towards caregiver					
Social support - FDW for general help	No	0.342 (0.180, 0.651)	0.001	0.342 (0.177, 0.658)	0.001
Social support - FDW for stroke patient	No				
Social Support (perceived)					
Care management strategies - Positive					
Care management strategies - Negative					
**STROKE PATIENT FACTORS**					
Age (in years)					
Gender	Male				
Ethnicity	Non-Chinese				
Marital Status	Single				
Ward Class	Unsubsidized				
CCI	1 - 3				
4 - 6					
≥7					
Stroke type	Non-ischemic				
Recurrent stroke	No	2.099 (1.136, 3.877)	0.018	2.097 (1.133, 3.880)	0.018
National Institute of Health Scale	Mild (0-4)				
Moderately severe (5-14)					

Severe (15-24)					
Modified Rankin Scale	No or slight disability (0-2)				
Moderate or severe disability (3-5)					
Mini-Mental State Examination	No cognitive impairment (24-30)				
Mild cognitive impairment (18-23)					
Severe cognitive impairment (1-17)					
Discharge to Community Hospital (step-down facility)	No				
Centre for Epidemiological Studies Depression Scale					

Abbreviations: aIRR: adjusted incidence rate ratio; CI: confidence interval; CCI: Charlson Comorbidity Index; FDW: foreign domestic worker

*: Model adjusted for age, gender, ethnicity and ward class of the patient

#: Model adjusted for age, gender, ethnicity, ward class of the patient and length of stay of index stroke hospitalization
